# Intelligent Vehicle Path Planning Based on Optimized A* Algorithm

**DOI:** 10.3390/s24103149

**Published:** 2024-05-15

**Authors:** Liang Chu, Yilin Wang, Shibo Li, Zhiqi Guo, Weiming Du, Jinwei Li, Zewei Jiang

**Affiliations:** State Key Laboratory of Automotive Simulation and Control, Jilin University, No. 5988, Renmin Street, Nanguan District, Changchun 130022, China; chuliang@jlu.edu.cn (L.C.); wyl22@mails.jlu.edu.cn (Y.W.); lisb22@mails.jlu.edu.cn (S.L.); zqguo23@mails.jlu.edu.cn (Z.G.); duwm21@mails.jlu.edu.cn (W.D.); lijw22@mails.jlu.edu.cn (J.L.)

**Keywords:** path planning, A* algorithm, intelligent driving, turning penalty function, obstacle raster coefficient

## Abstract

With the rapid development of the intelligent driving technology, achieving accurate path planning for unmanned vehicles has become increasingly crucial. However, path planning algorithms face challenges when dealing with complex and ever-changing road conditions. In this paper, aiming at improving the accuracy and robustness of the generated path, a global programming algorithm based on optimization is proposed, while maintaining the efficiency of the traditional A* algorithm. Firstly, turning penalty function and obstacle raster coefficient are integrated into the search cost function to increase the adaptability and directionality of the search path to the map. Secondly, an efficient search strategy is proposed to solve the problem that trajectories will pass through sparse obstacles while reducing spatial complexity. Thirdly, a redundant node elimination strategy based on discrete smoothing optimization effectively reduces the total length of control points and paths, and greatly reduces the difficulty of subsequent trajectory optimization. Finally, the simulation results, based on real map rasterization, highlight the advanced performance of the path planning and the comparison among the baselines and the proposed strategy showcases that the optimized A* algorithm significantly enhances the security and rationality of the planned path. Notably, it reduces the number of traversed nodes by 84%, the total turning angle by 39%, and shortens the overall path length to a certain extent.

## 1. Introduction

With the rapid development of intelligence and electrification in the automobile industry, autonomous driving technology plays a crucial role in modern traffic systems [1]. Path planning, as an important task for autonomous driving [2,3,4], can give the target vehicle priori information on the traffic network, contributing to driving safety and control robustness. However, the quality of the planning trajectory and the complexity of iterative calculation cannot be balanced well in practice when the algorithms face a complex traffic network environment [5,6,7]. Therefore, it is crucial to optimize and develop advanced path planning algorithms to improve the efficiency of path generation in various transportation network environments.

In the field of autonomous driving, particularly in robotic and UAV path planning, neural network technologies like cellular neural networks and global brainstorming have been widely applied [8]. For instance, in reference [9], by drawing an analogy between path planning and neural dynamics and propagating through the state space using neural dynamics, real-time collision-free planning was achieved. Its advantage lies in the lack of prerequisite information while treating target activity as an energy source, demonstrating parameter independence and physical validity. However, this method relies on complex neural dynamic models, requiring substantial computational resources to process data in real time. Reference [10] proposed a heat conduction method that establishes a transient heat conduction model and a cellular neural network to simulate heat conduction, generating the globally optimal path. Its strength lies in the natural avoidance of obstacles through heat conduction simulation. However, its transient model may be insufficiently responsive when dealing with dynamic environments. In the literature [11], an improved Hopfield-type neural network model is used to represent the local connectivity of the neural system as harmonic functions, propagating target activity via physical heat conduction. The advantage is that it allows for real-time obstacle avoidance without prior knowledge of dynamic environments. However, significant experimentation is needed to optimize model parameters. In reference [12], the global-best brainstorming optimization algorithm (GBSO) based on crossover recombination was designed to optimize paths with continuous curvature. This algorithm is particularly suitable for solving complex optimization problems with multiple constraints, but its efficiency is limited by the complexity of the constraints.

Automatic driving path planning algorithms can be categorized into two categories according to the generating principle of vehicle trajectory, including sampling-based and search-based trajectory planning algorithms [13,14]. To explore the principles and usage characteristics of different types of planning algorithms, this study conducted analyses on selected representative algorithms corresponding to the aforementioned two distinct planning principles. Table 1 shows a series of algorithms related to intelligent vehicle routing planning and their application scenarios.

In terms of the sampling-based algorithms [15,16,17], such as rapidly-exploring random trees (RRT) and probabilistic roadmap (PRM), always sample the vehicle state in the test space to find a feasible trajectory connecting the starting node and the goal node. RRT uses random sampling and the extension of the tree structure to find feasible paths in complex environments. It efficiently explores high-dimensional spaces and is suitable for real-time applications. However, it cannot find the globally optimal path due to its probabilistic nature as well as the sensitivity to parameter settings [18,19]. PRM constructs a road network by randomly sampling points in an obstacle environment to find the optimal path [20,21,22,23]. Nevertheless, constructing the network leads to burdensome computational intensity [24]. Random sampling cannot guarantee that the sample points cover the optimal path, posing a challenge to the efficiency of path generation in planning [25,26]. In conclusion, sampling-based algorithms can avoid local minima in non-convex spaces, and their advantage lies in their ability to handle planning problems in high-dimensional spaces and complex environments, demonstrating good adaptability. Despite the potential optimizations such as introducing heuristic functions, increasing sampling density, and improving connection strategies to address the computational challenges caused by the excessive sampling requirements of the sampling-based algorithm. In complex environments, the quality of paths planned by sampling-based algorithms is still influenced by the randomness in the distribution of sampling points.

In contrast to sampling-based approaches, search-based planning algorithms have the advantage of generating high-quality paths while reducing computational time and space complexity, such as Dijkstra’s algorithm and A* algorithm [27]. Dijkstra’s algorithm iteratively selects the vertex closest to the source point and updates the distances of its adjacent vertices using a priority queue [28]. The advantage of Dijkstra’s algorithm lies in its efficient ability to find the shortest paths from a single source point to all other vertices in the graph, provided that the edge weights in the graph are non-negative. However, a significant limitation of Dijkstra’s algorithm is its inability to handle graphs with negative edge weights, as the algorithm assumes that the shortest path is gradually constructed through vertices with the smallest known distances. The A* algorithm inherits the advantages of the Dijkstra algorithm, while improving search efficiency by introducing a heuristic function [29]. Nevertheless, it has some limitations in terms of heuristic function design and local optimal solutions, especially in the complex traffic environments encountered by unmanned vehicles [30]. Graph-based algorithms commonly face issues such as computational complexity and local optimum. The global nature of the search algorithm has led to challenges with insufficient computing resources and issues related to finding local optimal solutions [31]. Therefore, it is essential to address these issues through optimization techniques such as introducing parallel computing, refining heuristic functions, and enhancing algorithmic strategies. These measures aim to improve algorithm performance and mitigate the problem of local optima. Therefore, many experts and scholars, both domestically and internationally, mainly focus on improving the original algorithms and algorithm fusion orientation as the primary research directions in path planning [32,33,34,35].

An ideal method for autonomous driving path planning should be based on obstacle perception information, quickly and stably determining safe and reasonable paths, while considering their applicability to subsequent trajectory optimization and speed control [36]. The A* algorithm, as a classical heuristic search algorithm, has achieved significant success in the field of path planning [37]. However, when dealing with large-scale road networks, the traditional A* algorithm still faces challenges [38,39,40]. Researchers have proposed various methods to improve the global path quality in optimized path planning, addressing the shortcomings of a simple heuristic search, redundant control nodes, and low safety. Specifically, weighted heuristic functions, safety distance matrices, and key point selection strategies have been introduced into the traditional A* algorithm and integrated into the algorithm. In terms of the weighted heuristic function, a weighted heuristic function to enhance the computational speed of the A* algorithm is proposed in [41]. They retained only the turning points of the path and applied fifth-degree polynomial smoothing to effectively address the issue of path roughness. The method offers advantages such as improved computational speed, enhanced memory efficiency, and smoother paths. Nevertheless, due to the simplification of the search space, it cannot always yield the shortest path. Furthermore, its sensitivity to the initial heuristic function could lead to suboptimal solutions. In reference [42], the safety distance matrix was introduced into the A* algorithm, and the redundant nodes were removed from the path using a simplified method of separate terms. They also used arc transition at turning points to improve the safety and smoothness of the path. The advantages of this method include safer, smoother paths and higher computational efficiency. However, a fixed safety distance may not be suitable for complex environments or dynamic situations, and the removal of some nodes may lead to suboptimal paths. In addition, a key point selection strategy is proposed in the literature [43], which uses forced point-guided search to reduce the calculation of irrelevant points, deletes redundant points through secondary programming, and smooths dynamic tangent circles around the watershed. The advantages of this method include reduced computational time, better path smoothing, and further removal of redundant points. However, the processed path may be too close to obstacles, which is not safe, and it may also generate suboptimal paths due to the removal of some nodes.

In fact, in order to be more effectively applied to autonomous driving path planning, the improvement of the quality of traditional A* trajectories mainly faces three challenging problems, including the following:

(1) More absolute obstacle avoidance: In the face of the oblique arrangement and sparse occurrence of obstacles, the global path planned by the traditional A* algorithm will have the wrong path through the obstacles, which is a key problem affecting the feasibility of the planned path.
(2) Trajectory safety: The path nodes based on raster maps are often represented in the center of the grid, the distance between the nodes and the obstacles cannot be adjusted, and safety problems are caused by ignoring the safety distance constraint in the generated path.
(3) Concise control: The paths generated by traditional algorithms often have the problem of redundancy in control points, which leads to an unnecessary complexity of subsequent local optimization and control.

To optimize the A* path planning algorithm, some improvements mainly focus on reducing the number of traversed nodes, minimizing the total turning angle, incorporating safety distance, and shortening the path distance. In this context, an optimization strategy for A* path planning algorithm is proposed in this paper, aiming at getting the shortest global path based on reducing the number of traversed nodes and incorporating safety distance. To be specific, firstly, an optimized cost function based on the introduction of an obstacle raster coefficient and turn penalty function is introduced to enhance the adaptability and directionality of the search path to the map and minimize the total turning angle. Secondly, in order to solve the problem that the trajectory will pass through sparse obstacles, an efficient search strategy is designed and optimized to reduce the search space and computational complexity. Finally, the safety distance is integrated to improve the safety and self-adjustment performance of the path, and the redundant nodes are eliminated from the path data, and the control points and the total length of the path are greatly reduced, so that the path of the improved algorithm can take into account the requirements of efficiency and safety.

To enhance the performance of the A* algorithm and address its limitations in specific scenarios, this study introduces three core improvements aimed at optimizing the algorithm’s efficiency, safety, and applicability:

(1) By introducing an obstacle raster coefficient and a turning penalty function, this research develops a more accurate heuristic function. This function optimizes the cost calculation method as well as enhances the adaptability and directionality of the search path to map features, thereby improving the precision and practicality of path planning.
(2) In response to the issue of paths potentially traversing through sparse obstacles, this study has designed and optimized an efficient search strategy. By reducing the search space and computational complexity, this strategy significantly improves the reliability of path planning. It ensures that the planned paths avoid obstacles while also meeting the requirements for quick response.
(3) This research incorporates the concept of safety distance, substantially improving the safety and self-adjustment capabilities of the path. By eliminating redundant nodes from the path data and significantly reducing the number of control points and the total path length, an optimal balance between efficiency and safety in path planning is achieved.

The remainder of this study is organized as follows. The planning method of the traditional A* algorithm is provided in Section 2. Section 3 elaborates on the improvement process of the developed A* optimization algorithm. Section 4 discusses the simulation results and validates the superior performance of the raised strategy, followed by the main conclusions drawn in Section 5.

## 2. A* Algorithm Principle

**Figure 1:**
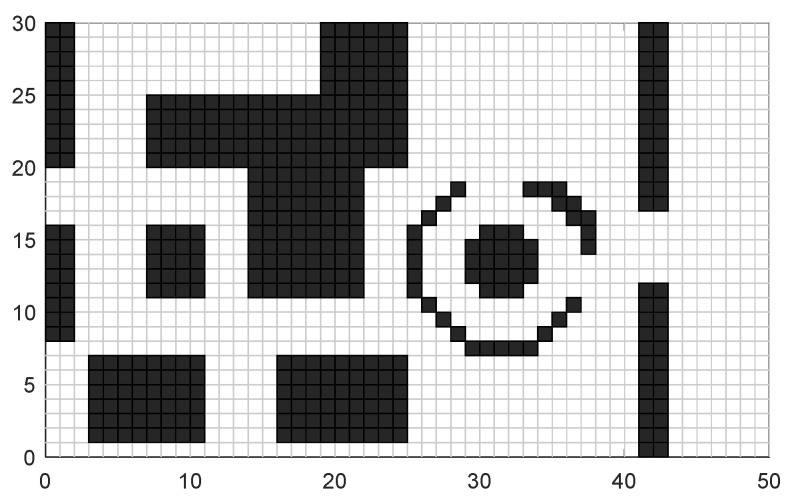
Raster test map.

The A* algorithm is a heuristic search algorithm that combines the Dijkstra algorithm and the greedy algorithm (depth-first). Its core concept is to find the minimum-cost path from the starting position to the target position in a specified global path map. Prior to planning, the A* algorithm requires a two-dimensional raster map for environment modeling. The environment information within the vehicle’s moving area in the world coordinate system is mapped onto a grid diagram. In Figure 1, a 30 × 50 grid map in the world coordinate system is depicted, which will serve as the test experimental map. The grid side length is denoted as “m = 1”, where the black areas represent obstacles, and the white areas signify feasible regions.

**Figure 2:**
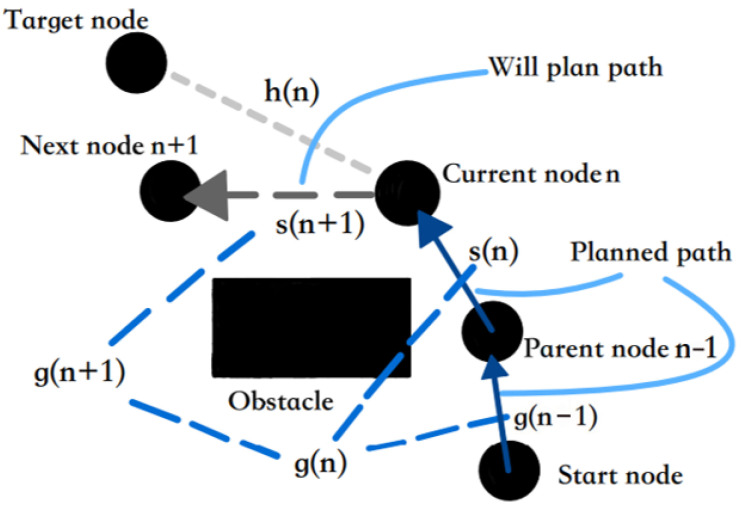
The schematic of A* search principle.

In the search process, the moving cost estimation function from the start position to the target position f(n) is defined as follows:

(1) f(n)=g(n)+h(n)

where, f(n) is the cost estimate from the initial state through state *n* to the target state, g(n) is the actual cost in the state space from the initial state to state *n*, and h(n) is the heuristic function of the best path from state *n* to the target state, that is, the estimated moving cost. The relationship between the planning node and the cost function in the search process is shown in Figure 2.

s(n) in Figure 2 represents the actual single-step moving distance from parent node *n* − 1 to current node n; s(n+1) represents the actual single-step moving distance from the current node n to the next node n + 1; and g(n) is the sum of the real moving distance g(n+1) from the start node to the *n* − 1 node and the actual single-step moving distance s(n) from the *n* − 1 node to the current node n, that is, the sum of the distance of each planned path. Thus, the expression of the true moving distance function g(n) can be given as:

(2) $$g(n)=\sum_{i=1}^{n}s(i)$$

If the evaluation value of h(n) is much less than g(n), then f(n) will be approximately equal to g(n). At this time, the algorithm A* is similar to Dijkstra algorithm, and the number of traversal nodes will increase, and the search efficiency will be greatly reduced. If h(n) is much larger than g(n), the A* algorithm gradually evolves into the best first search algorithm, and the path planning speed becomes faster, but the local optimal solution is prone to occur. Therefore, the performance of the A* algorithm depends on the selection of heuristic functions. Common estimation methods include Euclidean distance, Manhattan distance, and diagonal distance.

Suppose the starting point coordinates are $\left(s_{1},s_{2}\right)$, and the ending point coordinates are $\left(g_{1},g_{2}\right)$.

Then the Euclidean distance heuristic function is shown as follows:

(3) $$h=\sqrt{{\left(s_{1}-g_{1}\right)}^{2}+{\left(s_{2}-g_{2}\right)}^{2}}$$

The heuristic function of Manhattan distance is expressed as follows:

(4) $$h=\left|s_{1}-g_{1}\right|+\left|s_{2}-g_{2}\right|$$

The heuristic function of the diagonal distance is shown as follows:

(5) h=1.4×Diagonal+(Straight−2×Diagonal)

where,

(6) $$Diagonal=\min(\left|s_{1}-g_{1}\right|+\left|s_{2}-g_{2}\right|)$$

(7) $$Straight=\left|s_{1}-g_{1}\right|+\left|s_{2}-g_{2}\right|$$

Since the calculation accuracy of Euclidean distance is higher than that of Manhattan and diagonal distance, it is more likely to get the optimal path. Therefore, this paper chooses Euclidean distance as a heuristic function h(n) to predict the moving cost.

**Figure 3:**
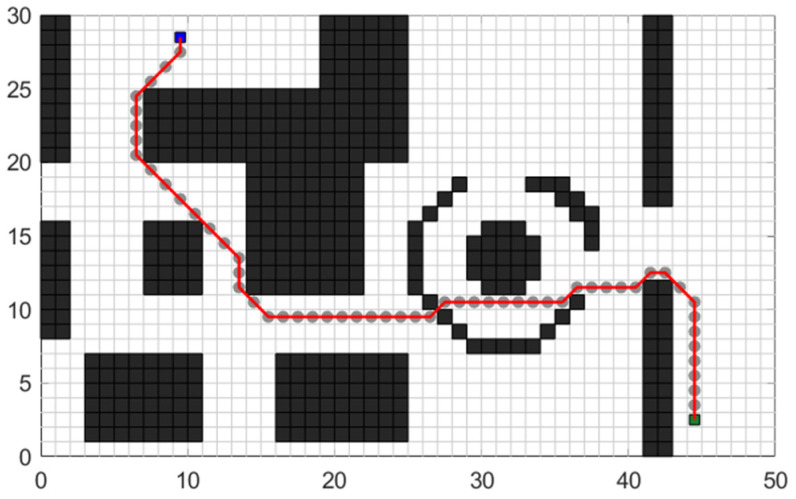
Planning trajectory of the traditional A* algorithm.

Based on the above algorithm principle, the path planning effect of the traditional A* algorithm displayed in the test map shown in Figure 1 is shown in Figure 3.

## 3. Optimization of A* Algorithm

**Figure 4:**
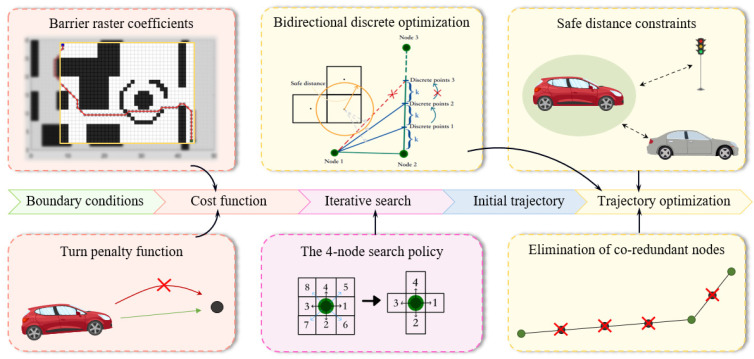
Schematic diagram of the technology route of optimization A* algorithm.

To address the issues encountered in the path planning process by the A* algorithm, such as simple heuristic search, redundant control nodes, and susceptibility to local optimality, this chapter focuses on three aspects of research: redesigning the cost function, improving the search strategy, and optimizing path nodes. This is illustrated in Figure 4, showcasing the enhanced overall technical pathway. Specifically, in order to improve the adaptability and heuristic performance of optimization algorithms in different planning scenarios, barrier raster coefficient and turn penalty function are integrated into the cost function. The barrier raster coefficient is defined to represent the distribution density of obstacles within the range restricted by the start and end points of the planning task. The turn penalty function steers the target vehicle towards searching in the direction of the shortest distance to the endpoint. As for the search strategy, a four-node search strategy is proposed to replace the original eight-node search strategy, overcoming the issue of the original algorithm path crossing between two obstacle vertices during searches. Finally, to enhance the safety and efficiency of the planned path, safety distance constraints are incorporated into the initial trajectory and redundant control nodes are removed through forward node detection and bidirectional discrete optimization.

### 3.1. Design of the Cost Function

#### 3.1.1. Barrier Raster Coefficients

In the traditional A* algorithm, the value of h(n) is crucial for global path planning, particularly in the early search process. Improving the heuristic can effectively reduce the number of nodes traversed by A* algorithm and its cyclic traversal speed, thereby enhancing search efficiency. However, when dealing with a path that encounters a large number of concave obstacles, an excessively large h(n) can disrupt the balance with g(n), leading the search step size to cross boundaries and resulting in a local optimal situation. To address this issue, based on the adaptive factor introduced in past research [44], the introduction form of the obstacle grid coefficient P was optimized to reflect the obstacle distribution in the planning process. This coefficient represents the density of obstacle grids in the local rectangular environment formed by the current node and the end point. The obstacle grid coefficient P provides map adaptability and does not require manual tuning. The formula is defined as follows:

(8) $$P=\frac{N}{\left(\left|x_{s}-x_{g}\right|+1)\times (\left|y_{s}-y_{g}\right|+1\right)}(P\in (0,1))$$

where, $\left(\left.x_{s},y_{s}\right)\right.$ are the coordinates of the start node, $\left(\left.x_{g},y_{g}\right)\right.$ are the coordinates of the target node, and N is the number of obstacle grids in the rectangular map formed by the cartesian coordinates of the start node and the target node.

The barrier grid coefficient P is incorporated into the heuristic function h(n) to get the modified heuristic function $h^{\prime }(n)$, which is shown as follows:

(9) $$h^{\prime }(n)=(1-\ln P)\cdot h(n)$$

where, (1−lnP) is the adaptive weight factor of the adaptive heuristic function $h^{\prime }(n)$. The obstacle raster coefficient P is introduced in the logarithmic form so that the heuristic function can also have a more sensitive adjustment effect on the sparse obstacle distribution. When the density of obstacles existing in the planning task is relatively large, the obstacle raster coefficient P is increased, and the adaptive coefficient (1−lnP) decreases accordingly, so that the modified heuristic function $h^{\prime }(n)$ decreases compared with the original heuristic function h(n) and the weight of g(n) is increased, so as to achieve a more reasonable obstacle avoidance effect.

The more obstacles in the map, the more the adaptive weight factor will decrease, and the proportion between g(n) and the heuristic function h(n) will be automatically adjusted to realize the adaptive adjustment of the cost function f(n), so as to avoid the local optimal situation and ensure the appropriate heuristic of the path planning and the map adaptability. The updated cost function f(n) is shown as follows:

(10) $$f(n)=g(n)+h^{\prime }(n)=\sum_{i=1}^{n}s(i)+(1-\ln P)\cdot h(n)$$

#### 3.1.2. Turn Penalty Function

The traditional A* algorithm may sometimes produce local optimal solutions, resulting in unnecessary turns in the planned path. Additionally, the path planning can exhibit turning redundancy due to search method limitations. To address these issues, a turn penalty function can be introduced to correct excessive corners in the path.

Firstly, the relative position from the parent node of the current node to the target node is determined, and the initial vector is obtained from the Cartesian coordinates of the two points. Next, the current node is connected to the target node to obtain the search vector. Finally, the position relationship between the initial vector and the search vector is determined using the principle of vector parallelism. Additionally, the turn penalty method is employed to align the searched path with the optimal path, thus minimizing unnecessary turns.

**Figure 5:**
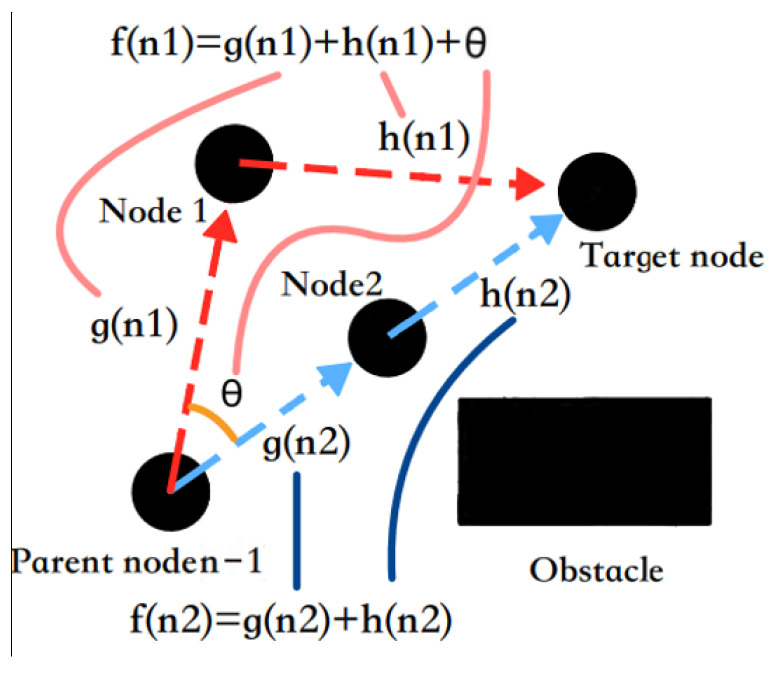
Turn penalty function schematic.

If the initial vector is parallel to the search vector, the path remains straight, and the turning cost is 0. Conversely, the turn cost function increases proportionally, aiming to minimize unnecessary turns in the planned path and align the search direction with the target node, ensuring global or relative optimization of the final path. The principle of path optimization based on the turn penalty function is illustrated in Figure 5. Nodes 1 and 2 in the figure are the child nodes of parent node n − 1. The size of the cost function of the search node after adding the turn penalty function is observed to be dependent on the direction vector of the child node and the target node. The turn penalty function is equal to 0 only when its direction aligns with the direction vector of its parent node pointing towards the target node. Ensure that the turn angle during each point search is always minimized.

Set the current node coordinates as $\left(n_{1},n_{2}\right)$, parent node as $\left(s_{1},s_{2}\right)$, and target node as $\left(g_{1},g_{2}\right)$; then, the direction vector can be described as follows:

(11) $$\left\{\begin{array}{l} dx_{1}=\left|g_{1}-n_{1}\right|,dy_{1}=\left|g_{2}-n_{2}\right|\\ dx_{2}=\left|g_{1}-s_{1}\right|,dy_{2}=\left|g_{2}-s_{2}\right| \end{array}\right.$$

According to the principle of vector parallelism, the expression of the turning penalty function is defined as follows:

(12) $$Turn\_penalty(n)=\left|dx_{1}\times dy_{2}-dx_{2}\times dy_{1}\right|\cdot K$$

where, K is the turning penalty coefficient.

The value of the turning penalty function is between 0 and 1, which is used to further improve the calculation accuracy of f(n). The updated cost function f(n) is shown as follows:

(13) $$\begin{array}{l} f(n)=g(n)+h^{\prime }(n)+Turn\_penalty\\ =\sum_{i=1}^{n}s(i)+(1-\ln P)\cdot h(n)+\left|dx_{1}\times dy_{2}-dx_{2}\times dy_{1}\right|\cdot K \end{array}$$

**Figure 6:**
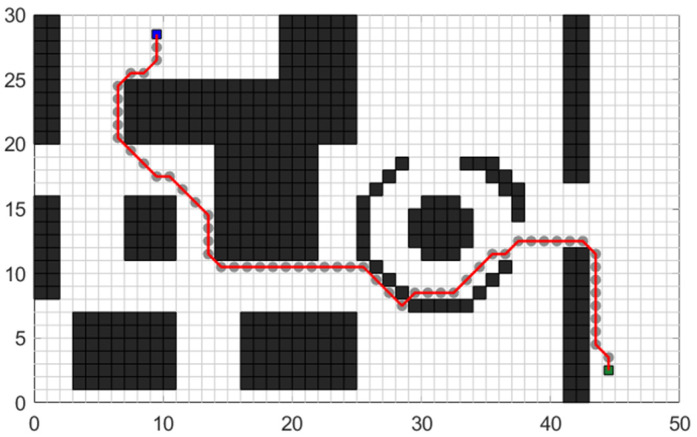
Planning trajectory after adding barrier raster coefficient and turn penalty function.

Figure 6 illustrates the path planning effect after introducing the barrier grid density P and the turning penalty function.

### 3.2. Search Policy Improvements

**Figure 7:**
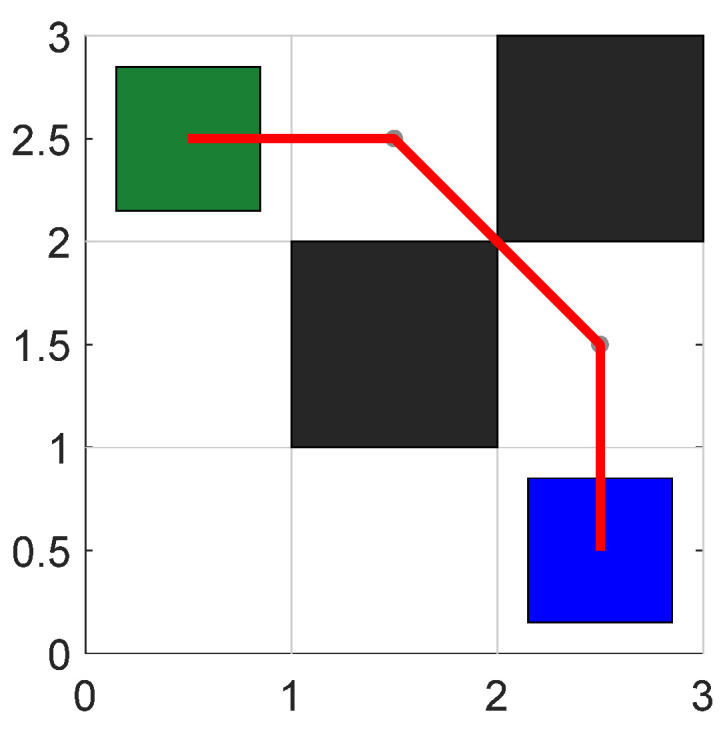
Diagram of the wrong condition where the path passes between two obstacles.

The traditional A* algorithm exhibits an unsatisfactory planning effect when dealing with an oblique arrangement and sparse distribution of obstacles, as depicted in Figure 7.

This collision problem significantly affects the feasibility of the planned path. The underlying reason for this problem is that the conventional eight-node search strategy of A* does not match well the appearance pattern of obstacles in the raster map. During the trajectory planning process, obstacles arranged diagonally are considered passable at the vertices of the grid, leading to collisions between the planned trajectory and the obstacles.

**Figure 8:**
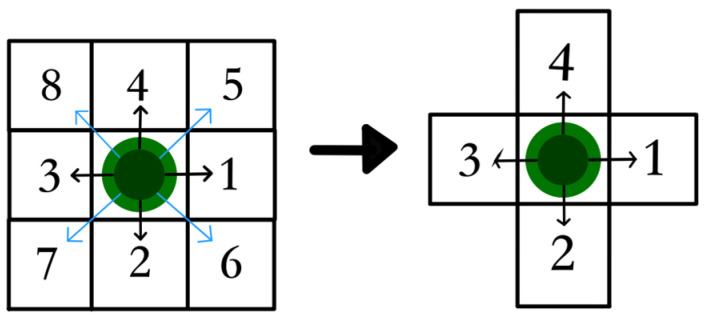
Schematic diagram of replacing the search policy.

To overcome the collision problem, this paper analyzes and simplifies the algorithm’s search strategy, introducing a novel search point selection strategy. This strategy excludes certain suboptimal child nodes, and the traditional eight-node search strategy is transformed into a more efficient four-node search strategy, as illustrated in Figure 8.

In the two-dimensional Euclidean space, the traditional A* algorithm expands the surrounding eight nodes in each search process, as illustrated in the left diagram of Figure 8. Using a two-dimensional vector, the movement towards the eight nodes from the current node can be represented. For vector $\vec{a}=\left(a_{1},a_{2}\right)$, the calculation formula for its Euclidean norm is shown as follows:

(14) $${\left\|\vec{a}\right\|}_{2}={\left(\sum_{i=1}^{2}{\left|a_{i}\right|}^{2}\right)}^{\frac{1}{2}}$$

Based on the magnitude of the Euclidean norm, the eight vectors can be divided into two groups, nodes 1–4 and nodes 5–8, representing two different search step lengths, as shown in the following:

(15) $$\left\{\begin{array}{l} {\left\|\vec{a}\right\|}_{2}={(\sum_{i=1}^{2}{\left|a_{i}\right|}^{2})}^{\frac{1}{2}}=\sqrt{1^{2}+0^{2}}=1,\mathrm{node}\;1-4\\ {\left\|\vec{a}\right\|}_{2}={\left(\sum_{i=1}^{2}{\left|a_{i}\right|}^{2}\right)}^{\frac{1}{2}}=\sqrt{1^{2}+1^{2}}=\sqrt{2},\mathrm{node}\;5-8 \end{array}\right.$$

To avoid collisions with diagonally arranged obstacles, the forward movement options for nodes 5–8 are excluded, retaining nodes 1–4, which maintain the search step of 1. This transition changes the search strategy to a four-node search strategy, as shown in the right image in Figure 8.

**Figure 9:**
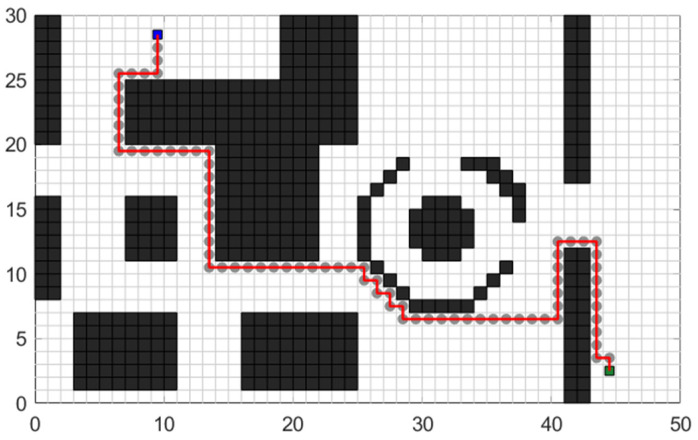
Planning trajectory after improving the search strategy.

The comparison of the improved search strategy’s effect on path planning is presented in Figure 9. This approach not only reduces the search time and scope for each step, thereby enhancing running speed, but also effectively addresses the mismatch between the search method and the raster map. By avoiding paths that pass through the vertices of obstacles, the likelihood of collisions between the driverless car and obstacles is reduced. However, this optimization Inevitably results in some negative impacts, including an increase in the number of turns, traversed nodes, and path length. Nonetheless, these negative effects are confined to a small and concentrated range, which will be addressed in the subsequent third part of path optimization.

### 3.3. Optimization of Path Nodes

#### 3.3.1. Safe Distance Constraints

The traditional A* algorithm restricts path nodes to be located at the center of the grid, resulting in the distance between the path nodes and obstacles being solely determined by the precision of the grid map. Specifically, when the path passes through grid vertices, the distance between the path and obstacles is 0, which leads to insufficient safety in path planning. Therefore, other research [45] has detected nodes with obstacles around them and moved the node to a grid in the opposite direction to achieve the purpose of the principled obstacle. On this basis, the improved A* algorithm incorporates the constraint of a safe distance, offsetting the path nodes to positions that are sufficiently safe from obstacles.

In the case of a planning problem in two-dimensional Euclidean space, where the planned path consists of n sequential connections of two-dimensional nodes, the i-th path node $\sigma_{i}$ can be represented as follows:

(16) $$\sigma_{i}=(\sigma_{ix},\sigma_{iy})\in {\mathbb{R}}^{2\times 1},i=0,\cdots ,n$$

For the traditional A* algorithm, after setting the starting point S and the target point T, the global planning result $\Gamma_{ST}$ obtained through the search can be represented as follows:

(17) $$\Gamma_{ST}={\left(\sigma_{0},\sigma_{1},\cdots ,\sigma_{n-1},\sigma_{n}\right)}^{T}\in {\mathbb{R}}^{n\times 2}$$

where $\sigma_{0}=S,\sigma_{n}=T$.

Similarly, the set of obstacles can be represented as follows:

(18) $$\Phi ={\left(\varsigma_{0},\varsigma_{1},\cdots ,\varsigma_{m-1},\varsigma_{m}\right)}^{T}\in {\mathbb{R}}^{m\times 2}$$

Taking the *i*-th path node $\sigma_{i}$ as an example, find the obstacle node $\varsigma_{j}$ that is closest to the path node and calculate the distance between the obstacle grid center and the path node, as shown in the following:

(19) $$sd(\sigma_{i},\varsigma_{j})=\min{\left\|\vec{\left(\sigma_{i},\varsigma_{j}\right)}\right\|}_{2},j=0,\cdots ,m$$

The safe distance represents the minimum required distance between the path control points and obstacle edges. A safety distance evaluation function is used to assess each control path point, and the path points are adjusted individually to obtain a safe path that meets the safety requirements while avoiding unnecessary redundant paths caused by the vehicle being far away from obstacles. The safety distance judgment function and adjustment strategy are represented as the following:

(20) $$\left\{\begin{array}{l} \sigma_{ix}=\sigma_{ix}-\left(safe\_dist-\frac{d}{2}\right),sd(\sigma_{i},\varsigma_{j})≺safe\_dist+\frac{d}{2}\&\varsigma_{ix}=\sigma_{ix}+1\\ \sigma_{ix}=\sigma_{ix}+\left(safe\_dist-\frac{d}{2}\right),sd(\sigma_{i},\varsigma_{j})≺safe\_dist+\frac{d}{2}\&\varsigma_{ix}=\sigma_{ix}-1\\ \sigma_{iy}=\sigma_{iy}-\left(safe\_dist-\frac{d}{2}\right),sd(\sigma_{i},\varsigma_{j})≺safe\_dist+\frac{d}{2}\&\varsigma_{iy}=\sigma_{iy}+1\\ \sigma_{iy}=\sigma_{iy}+\left(safe\_dist-\frac{d}{2}\right),sd(\sigma_{i},\varsigma_{j})≺safe\_dist+\frac{d}{2}\&\varsigma_{iy}=\sigma_{iy}-1\\ \sigma_{i}=\sigma_{i},sd(\sigma_{i},\varsigma_{j})\geq safe\_dist+\frac{d}{2} \end{array}\right.$$

**Figure 10:**
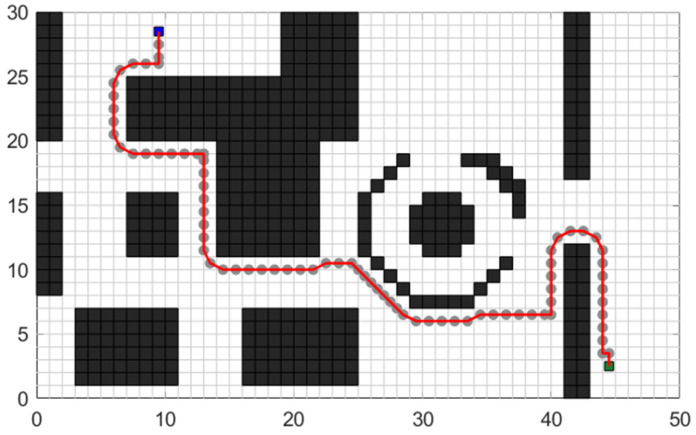
The effect of the path point offset.

According to Equation (20), each path node can be offset based on the safety distance constraint. The path planning results considering the safe distance and waypoint offset handling are shown in Figure 10.

#### 3.3.2. Extraction of Necessary Nodes

Based on the current improvements, there are still issues regarding excessive traversal nodes and redundant turning points. These aspects can result in numerous unnecessary control points during actual driving, leading to higher vehicle energy consumption. To address this problem, this section proposes the elimination of redundant nodes in the same direction. Meanwhile, obstacle collision detection and bidirectional discrete smoothing optimization are integrated to reduce the number of turning points and the overall length of the path, thereby enhancing the smoothness of the planning outcome.

(a) Elimination of co-redundant nodes

When the vehicle is moving in a straight line, the intermediate nodes used for controlling the driving direction become redundant. To eliminate these unnecessary nodes in the same direction, a same direction detection method is proposed for the current path nodes. A set of direction vectors are generated by using three adjacent nodes. If the cross product result of the two vectors is zero, it indicates that the three points are collinear, and the central redundant node should be eliminated. Conversely, if the result is non-zero, the central node is essential and should be retained as a new starting point. By repeating this process, all of the same direction redundant nodes are eliminated, thus reducing the number of control points.

Suppose that the coordinates of the three consecutive points of the planned path are $A(x_{1},y_{1})$, $B(x_{2},y_{2})$, and $C(x_{3},y_{3})$, and direction vectors can be written as follows:

(21) $$\left\{\begin{array}{l} \vec{AB}=(x_{2}-x_{1},y_{2}-y_{1})\\ \vec{AC}=(x_{3}-x_{1},y_{3}-y_{1}) \end{array}\right.$$

The cross product of the two vectors is written as follows:

(22) $$\vec{AB}\times \vec{AC}=(x_{2}-x_{1})\cdot (y_{3}-y_{1})-(x_{3}-x_{1})\cdot (y_{2}-y_{1})$$

**Figure 11:**
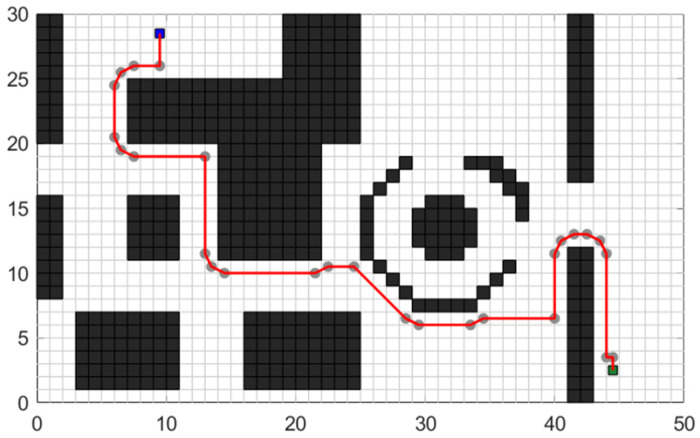
Planning trajectory after eliminating redundant nodes in the same direction.

If $\vec{AB}\times \vec{AC}=0$, point B is abandoned, point C is retained and the test continues with point C as the vector starting point; if $\vec{AB}\times \vec{AC}\neq 0$, point B is retained and the test continues with point B as the vector starting point. The path planning effect after the elimination of redundant nodes in the same direction is shown in Figure 11.

(b) Bidirectional discrete optimization

To further optimize the path points and address the issue of redundant paths caused by nodes being only in the center of the grid, this study proposes a combination of obstacle collision detection and bidirectional discrete smoothing optimization. First, analyze each line segment of the current path and discretize it to obtain a series of discrete path control points separated by a small interval K. Each discrete point is then reconstructed, and obstacle collision detection is performed in combination with the safety distance. The goal is to find the minimum turning path that meets the safety requirements.

**Figure 12:**
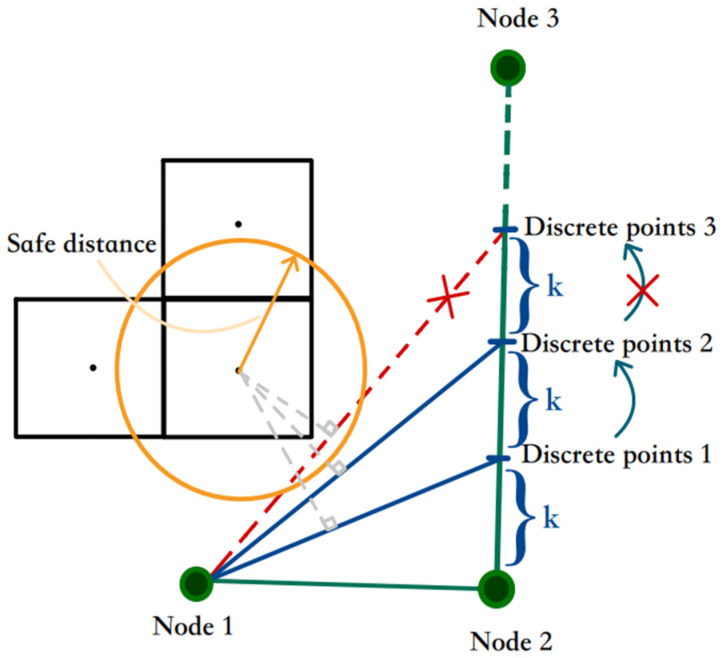
The schematic of discrete smoothing optimization.

Figure 12 illustrates the post-discrete collision detection of a single line segment in the bidirectional discrete smooth optimization process. The green path represents the path to be optimized, and the discrete points represent a series of dense points along the path. The obstacle grid center nearest to each line segment is identified to compare the distance between the vertical segment from the center to the line segment with the safety distance. If the safety requirements are met, repeat the process for the next line segment. When the line segment is cut from the safe distance circle, the previous discrete point is regarded as a necessary inflection point. Then connect it with the discrete point of the next line segment to form a new node, and repeat the process until reaching the target node. This process is performed in each direction to maximize the approximation of the optimal path planning and reduce the number of traversing nodes, thus compensating for the negative optimization effect mentioned in Section 3.2.

**Figure 13:**
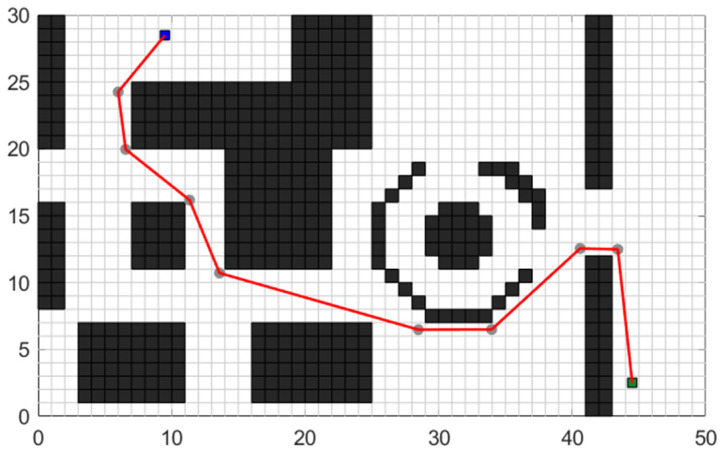
Test effect of the optimized A* algorithm.

At this point, the three-layer optimization process of traditional A* has been completed, and the final path planning effect is shown in Figure 13.

### 3.4. The Flow of A* Optimization Algorithm

To enable the A* optimization algorithm to plan the optimal path in complex maps, effectively reducing the number of traversing nodes, total turning angle, search speed, and path distance, this study integrates three strategies: the adaptive heuristic cost function strategy, the improved neighborhood priority search strategy, and the redundant node smoothing strategy. These strategies culminate in the proposed improved A* algorithm presented in this paper. The algorithm pseudocode is depicted in Table 2.

## 4. Testing & Evaluation

### 4.1. No Obstacle Test Results

**Figure 14:**
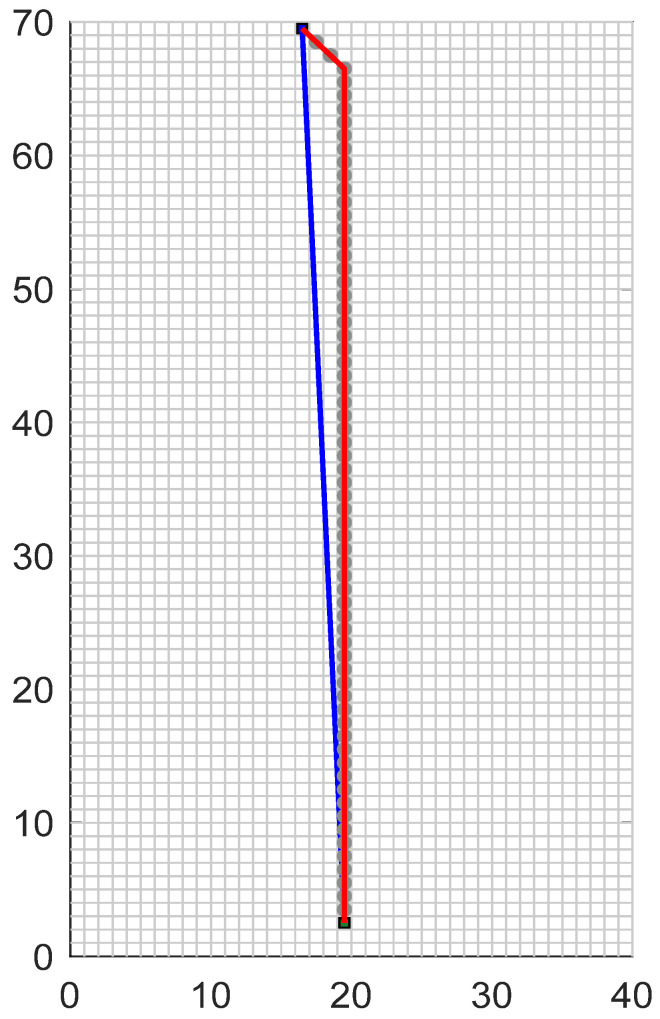
Comparison of traditional A* algorithm and A* optimization algorithm on maps without obstacles.

In order to explore the performance benchmark of the optimized A* algorithm compared with the traditional A* algorithm in ideal conditions, a 40 × 70 raster test scenario is established, and the planning effect of the optimized algorithm on an accessible raster map is shown in Figure 14.

As shown in Figure 14, the red line represents the planning path of the traditional A* algorithm under the barrier-free object map, and the blue line represents the planning path of the optimized A* algorithm under the barrier-free object map. Due to the improved A* algorithm with a series of optimization processes such as discrete smoothing optimization, the optimized A* algorithm plans a more reasonable and smooth global path in the face of the map without obstacles. The performance indicators of the algorithm before and after the improvement in path planning are compared in Table 3.

As shown in Table 3, the optimized A* algorithm has greatly improved the number of control nodes and the total turning angle, and has reached the shortest distance between the starting and ending points in terms of the total length of the path. In summary, the optimized A* algorithm shows more significant planning advantages than the traditional A* algorithm in the barrier-free map environment.

### 4.2. Establishment of Unstructured Test Environment

**Figure 15:**
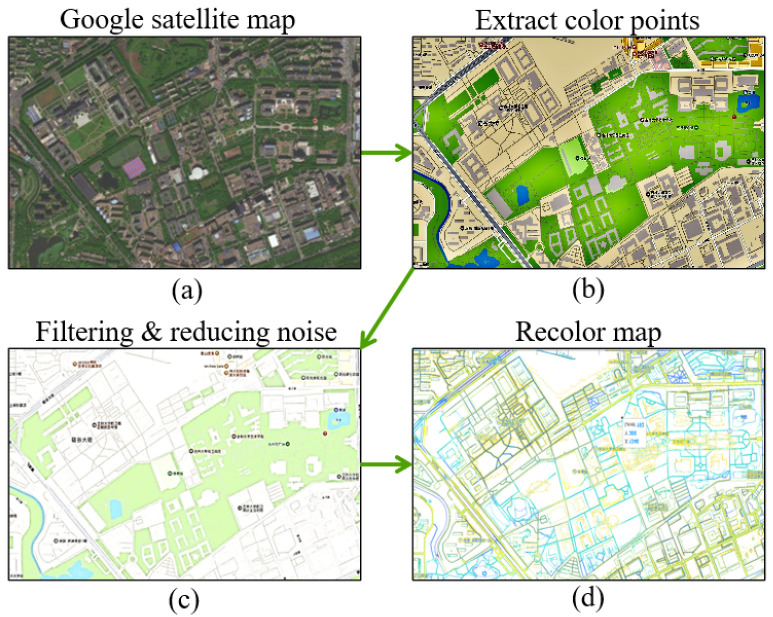
The extraction process of satellite maps. Figure (**a**) represents the satellite map of the testing environment, figure (**b**) illustrates the image after extracting color points from the original map, figure (**c**) depicts the image after processing with a filtering and noise reduction function, and figure (**d**) shows the re-colored map image.

To verify the optimization effect of the traditional A* algorithm in an unstructured environment, the real satellite map image of the South District of Jilin University was acquired by using Google Map, as illustrated in Figure 15a. The map satisfies the testing requirements for global path planning algorithms in the unstructured environment in terms of its area, large number of buildings, and clear division of feasible areas. Additionally, it meets the requirements for mapping a raster map. To create a suitable environment for testing, moving pedestrians and vehicles were excluded. Figure 15b–d shows the image after filtering and reducing noise, as well as the re-coloring process to extract color points from the map.

**Figure 16:**
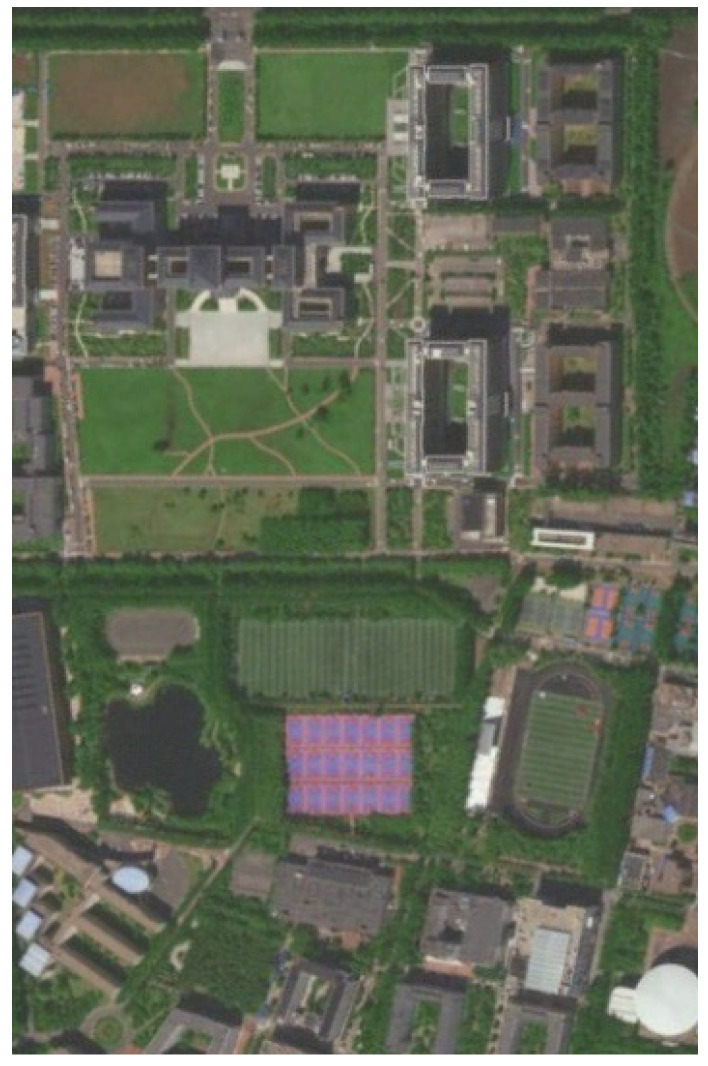
Intercepted satellite map.

**Figure 17:**
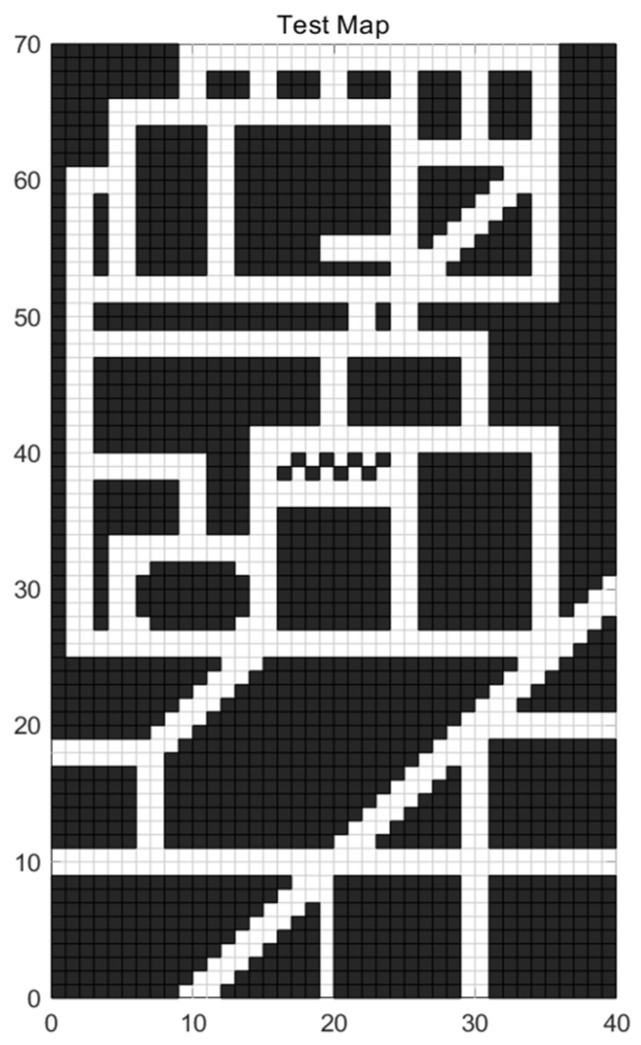
Raster map of the avant-garde south area of Jilin University.

Based on the processing results, a portion of the region with a better recognition effect was selected to create a raster map for the optimization effect test platform. Figure 16 shows the satellite map of the selected area, while Figure 17 displays the corresponding raster map which is established from this captured part.

### 4.3. Display of Unstructured Test Results

**Figure 18:**
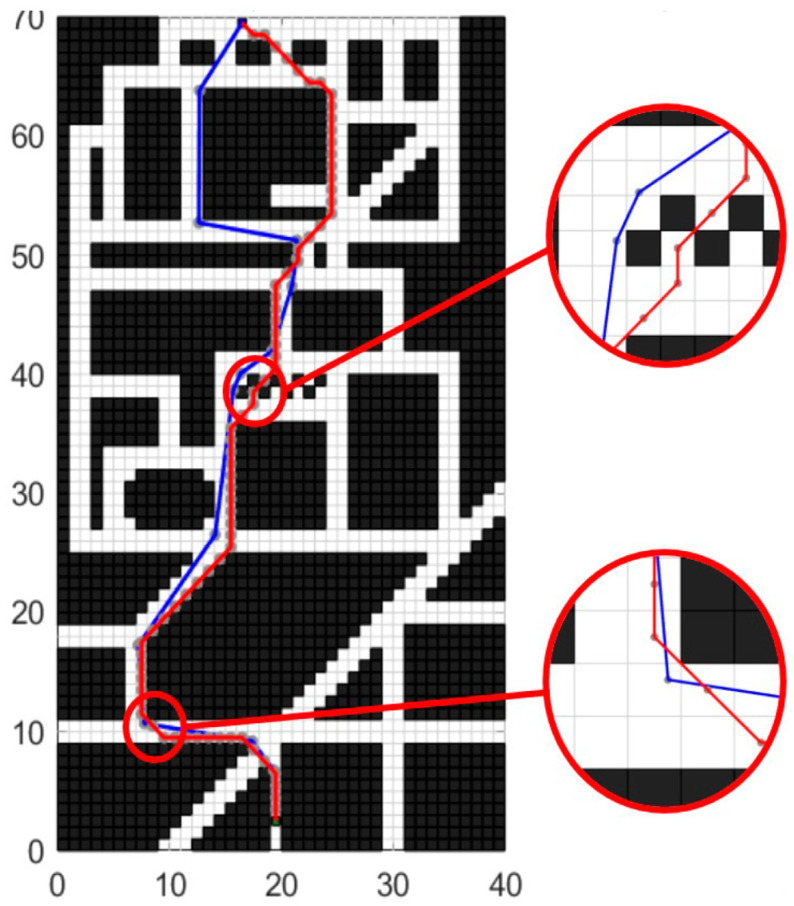
Comparison of traditional A* algorithm and A* optimization algorithm in an unstructured map.

The experimental scenario involved a path planning problem from the entrance to the school gate to the apartment building. This scenario was mapped to the raster map, and the starting point coordinates were set as (17, 70), while the end coordinates were set as (20, 3). The path planning effects of the improved A* algorithm before and after optimization are illustrated in Figure 18.

In Figure 18, the red line represents the planned path using the traditional A* method, while the blue line represents the path planning effect using the improved A* method. The local enlarged image in the figure demonstrates that the improved A* algorithm can effectively avoid the incorrect planning of the path passing through the gap of the grid while maintaining a certain safe distance from obstacles. Additionally, it retains the advantage of the traditional A* algorithm in always searching in the shortest direction of the path. The performance indicators of the algorithm before and after the improvement in path planning are compared in Table 4.

As shown in Table 4, the optimized A* algorithm significantly reduces the number of traversed nodes and the total turning angle of the path, while also considering the safe distance from obstacles. This simplifies the control points, reduces turning, and improves energy consumption, driving experience, and driving safety. Although the total path length increases compared to that of traditional planning methods, the local enlarged figure indicates that the optimized algorithm employs reasonable obstacle avoidance planning to prevent the wrong “through the wall” type of path planning that the traditional algorithm may produce. This results in a notable increase in the path length of the optimized algorithm in this case. However, this increase is higher than the difference in the total planned path lengths shown in Table 4. Thus, the optimized A* algorithm still outperforms the traditional algorithm in reducing the total path length. As for the planning time, the optimized A* algorithm requires an additional traversal of the initial trajectory due to the presence of a discrete smoothing optimization process, resulting in a longer planning time. However, the global planning process typically occurs only at the beginning of autonomous driving tasks or when the vehicle significantly deviates from the planned trajectory and requires replanning. Additionally, global paths with fewer control nodes will significantly reduce the time required for backend trajectory optimization. Therefore, the sacrificed planning time by this optimization algorithm is acceptable.

**Figure 19:**
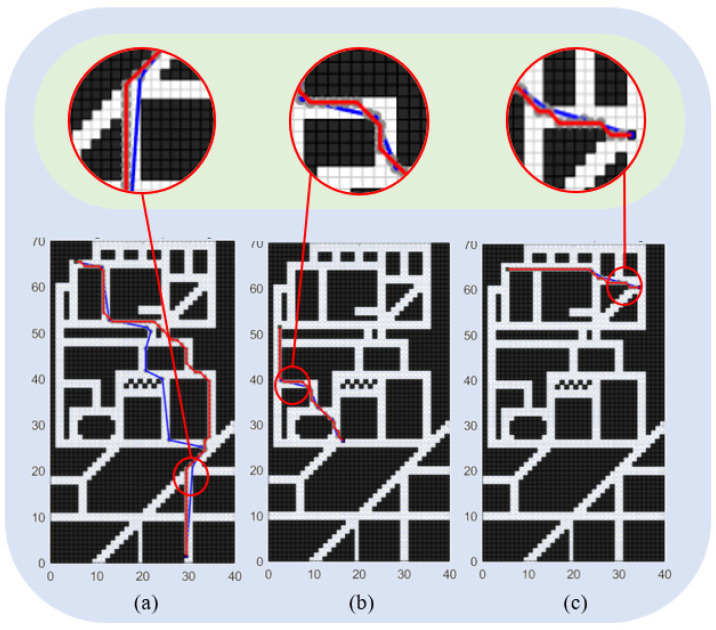
Robustness test effect of optimized A* algorithm: (**a**) shows the planning result in a long-distance environment, (**b**) shows the planning result in a medium-distance environment, and (**c**) shows the planning result in a short-distance environment.

As depicted in Figure 19, several tests are conducted with different starting and ending coordinates to evaluate the robustness and efficiency of the improved algorithm in handling various complex road conditions and planning requirements. Figure 19a, Figure 19b, and Figure 19c, respectively, illustrate the planning performance of the enhanced A* algorithm in long-distance planning tasks, medium-distance planning tasks, and short-distance planning tasks. The three insets provide a detailed magnification of the local regions, demonstrating that compared to the traditional A* algorithm, the enhanced A* algorithm exhibits superior efficiency and rationality in planning paths across three different scales of planning tasks. The planning results demonstrate that the improved algorithm performs consistently well across different scenarios.

Table 5 provides specific performance indicators for each test case. It shows that the optimized A* algorithm maintains its effectiveness in reducing the number of traversal nodes and the total turning angle, while ensuring a safe distance from obstacles. The algorithm’s ability to simplify control points, minimize turning, and improve energy consumption and driving safety remains evident in all test scenarios.

As shown in Table 5, when the algorithm is provided with three different planning requirements, namely short, medium, and long paths, the improved A* algorithm demonstrates significant optimization compared to the traditional A* algorithm. Across all three scenarios, the improved A* algorithm consistently plans shorter paths, reduces cumulative turning angles, minimizes the number of traversing nodes, and maintains a safe distance from obstacles.

In summary, the optimized A* algorithm exhibits remarkable path planning efficiency and satisfactory performance indices in comparison to the pre-optimized version. Its enhanced robustness and adaptability enable it to handle diverse path conditions with varying planning requirements. The improved A* algorithm represents a valuable contribution to the field of path planning, offering a reliable and effective solution for optimizing vehicle trajectories in complex environments.

### 4.4. Exploration of the Influence of Obstacle Grid Coefficient P on Planning

**Figure 20:**
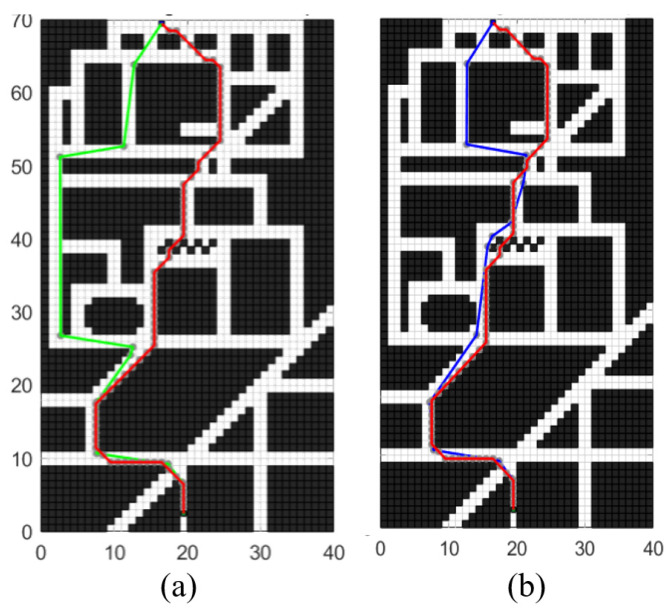
Comparison of the planning effect of the dynamic obstacle raster coefficient P: (**a**) shows the optimization effect under dynamic adjustment parameter P, and (**b**) shows the optimization effect under static parameter P.

To further explore the impact of obstacle grid coefficient P on planning effectiveness, this section attempts to change the determination of P from being determined by the start node and target node to being determined by the current node and target node, aiming to achieve dynamic parameter adjustment. In the unstructured grid map environment shown in Figure 17, the starting point coordinates were set as (17, 70), while the end coordinates were set as (20, 3). A comparative verification test was conducted on the improved algorithm before and after modification, with the test results shown in Figure 20.

In Figure 20a, the green line in the graph represents the testing result after dynamically adjusting P, while the red line represents the planning result of the traditional A* algorithm. In Figure 20b, the blue line depicts the testing result under a constant P, while the red line represents the planning result of the traditional A* algorithm. It can be observed that under the dynamically adjusted parameter P, the planning algorithm tends to choose routes that are farther but with lower obstacle density. To further compare the effects of setting P as a dynamic parameter or a constant term on planning effectiveness, Table 6 presents simulation data for the three methods.

As shown in Table 6, the paths planned with the updated obstacle grid coefficient P exhibit fewer control nodes, yet they also demonstrate longer path lengths and increased total turning angles. This indicates suboptimal optimization outcomes. Consequently, the measure of altering the starting node to the current node for dynamic parameter adjustment enables each search iteration in the planning process to select more open paths based on the obstacle density ahead, resulting in a superior number of control nodes. However, in the test maps utilized in this study, this adjustment leads to an increase in both the total path length and total turning angles.

In summary, the obstacle grid coefficient P is intended to represent the obstacle density between two nodes, thereby allowing the planning direction to be adjusted based on the density of obstacles ahead, enhancing the heuristic nature of the search process. In this study, P is fixed as a constant determined by the starting and target nodes to adjust the ratio of weights between g and h in the heuristic function, making the heuristic search process more adaptive and heuristic, aiming to obtain the shortest path in unstructured scenarios. However, adjusting P as a dynamic parameter tends to plan paths with fewer obstacles and more open roads, which is suitable for some planning scenarios in structured road networks.

### 4.5. Exploration of the Influence of Turning Penalty Function on Planning

**Figure 21:**
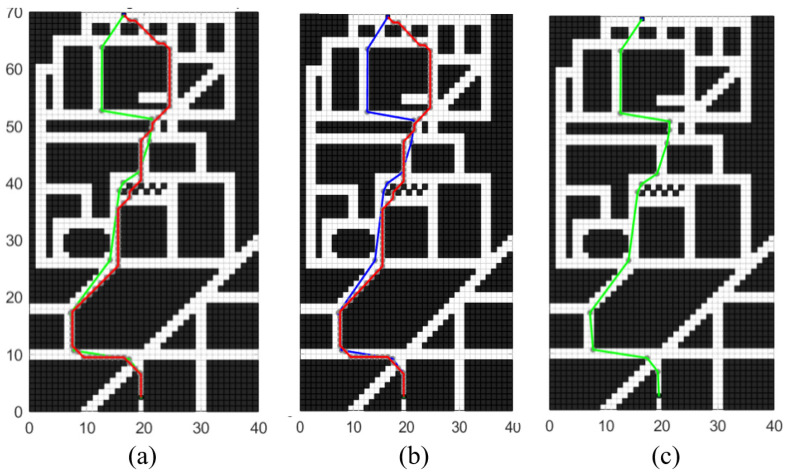
Comparison of the planning effect of adjusting the turning penalty function: (**a**) shows the optimization effect without the turning penalty function, (**b**) shows the optimization effect with the turning penalty function, and (**c**) shows two paths of adjusting the turning penalty function.

To further explore the impact of turning penalty function on planning effectiveness, this section tries to remove the turning penalty function and compare it with the previous planning effect in the unstructured grid map environment shown in Figure 17; the starting point coordinates were set as (17, 70), while the end coordinates were set as (20, 3). A comparative verification test was conducted on the improved algorithm before and after modification, with the test results shown in Figure 21.

As shown in Figure 21a, the green line represents the testing performance after removing the turn penalty function, while the red line denotes the planning performance of the traditional A* algorithm. In Figure 21b, the blue line illustrates the testing performance with the inclusion of the turn penalty function, juxtaposed with the red line depicting the planning performance of the traditional A* algorithm. Figure 21c presents a schematic diagram of the planning paths concurrently retaining both the blue and green lines. It is observable that their trajectories completely overlap, indicating that in this study, incorporating the turn penalty term into the cost function does not affect node selection. This is because the magnitude of the turn penalty function depends on the angle of the deviation of the search node. In the neighborhood node search mode of the A* algorithm, the angle of node deviation is proportional to the distance between the node and the target node. Therefore, in the cost function f, the turn penalty function and the h term increase or decrease simultaneously, achieving the same node selection effect. However, the turn penalty function, as a factor proportional to the deviation direction of the path, can be beneficial in specific planning scenarios, directing the path towards a direction more favorable to the target node. Consequently, this facilitates a quicker approach of the path towards the target node, leading to a more significant heuristic effect.

## 5. Conclusions

In this paper, an optimized A* algorithm is designed for global path planning, building on the traditional A* algorithm. Key findings include the following:

1. The heuristic function is refined by the obstacle raster coefficient and the turning penalty function. This enhancement improves the adaptability and directionality of the search path relative to terrain features, avoiding sharp turns and efficiently navigating around obstacles.
2. The design of efficient search strategy addressed the problem of planning the path through sparse obstacles. By strategically reducing the search space and computational complexity, this approach has significantly enhanced the algorithm’s performance.
3. The optimization of the initial path nodes ensures a necessary safety margin from obstacles while significantly reducing the number of path nodes, effectively balancing the objectives of safety and efficiency.

The limitations are expressed as follows:

1. The performance of the optimized algorithm in extremely complex environments or under conditions of limited computational resources remains a challenging frontier.
2. The study lacks the capability to handle dynamic obstacle environments and overlooks the constraints related to dynamic obstacle avoidance.

Looking forward to the future, future research work will develop a backend local trajectory optimization algorithm based on the rough initial path obtained in this algorithm, combined with vehicle dynamics constraints, static obstacle avoidance constraints, and dynamic obstacle avoidance constraints, and explore the performance of the algorithm in real-world scenarios with dynamic and unpredictable obstacles. It aims to plan a trajectory planning algorithm that is more efficient, safer, and easy for vehicles to smoothly follow.

## Figures and Tables

**Table 1:** The algorithms of intelligent vehicle planning.

Algorithm	Algorithm Type	Characteristic	Application Scenarios
PRM	Sampling-based	After uniform sampling, the path selection is realized based on A*	The target point needs to be stable and have certain environmental prior information.
RRT		Random tree expansion with probability completeness to ensure path optimality	Tree node extension sampling determines that it is suitable for global path planning.
Dijkstra	Search-based	Breadth-first search	It is often used to solve the problem of shortest path for a single source, such as in network routing and urban transportation planning.
A*		Heuristics enable a fast directed search	Unable to handle dynamic and random obstacles

**Table 2:** The pseudocode of improved A* algorithm.

Improved A* Algorithm	
Input: Environment Data **A**, Start Node S, Target Node T, Safe Distance SD, Dispersion t	
Output: Optimized Path **OP**	
1:	$A=\left(\begin{array}{ccc} a_{11} & \ldots & a_{1n}\\ \vdots & \ddots & \vdots \\ a_{m1} & \cdots & a_{mn} \end{array}\right);ai_{j}\in [0,1]$ // Quantify environment information to **A**
2:	E=rasterize(A); // Rasterize map based on **A**
3:	P=barrier_coef(E,S,T); // Calculate barrier raster coefficient P using S and T
4:	Add S to **IP**; // Initialize path **IP** with S
5:	CN = S; // Set current node CN to S
6:	while CN ≠ T // Terminating criteria for the search
7:	EN = 4node_mode (CN, E); // Finding neighborhood nodes
8:	$f_{EN}=\alpha \cdot P+g(EN)+h(EN)+TP(EN);$ // Recalculate f using cost function CF
9:	$CN=\underset{EN}{\min}\;f_{EN};$ // Set CN to the node with smallest f in O
10:	Add CN to **IP**; // Add to initial path **IP**
11:	end;
12:	$IP=(S,p_{1},\cdots ,p_{k-1},T);p_{i}\in {\mathbb{R}}^{2}$ // Retrieve initial path **IP** from S to T
13:	SP=safe_dis(IP,SD,E); // Apply SD to **IP** to get safe path **SP**
14:	RP=co_eli(SP); // Remove redundant nodes from **SP** to get **RP**
15:	DP=discrete(t,RP); // Discretize the **RP** route with discreteness t
16:	while j ≠ T // i,j∈DP;
17:	if collision_dete(i,j,E)=false // Collision detection
18:	i = j;
19:	Add i to **OP**; // Node i is necessary, add it to result path **OP**
20	end;
21:	j = j + 1;
22:	end;
23:	Repeat the process of lines 17–23 in the opposite direction
24:	Return **OP**;

**Table 3:** Performance indicators of planning algorithms.

Test Items	Traditional A* Algorithm	A* Optimization Algorithm	Performance Optimization Rate
Path nodes	68	2	97.1%
Total turning angle (°)	45	0	100.0%
Path length (m)	1023	1006	1.7%

**Table 4:** Performance indicators of planning algorithms.

Test Items	Traditional A* Algorithm	A* Optimization Algorithm	Performance Optimization Rate
Path nodes	75	12	84.0%
Safe distance (m)	0	1	-
Total turning angle (°)	855.1	522.3	39.0%
Planning time (ms)	709.2	986.6	−39.1%
Path length (m)	1314	1332	−1.4%

**Table 5:** Robustness test performance indicators.

Test Conditions	Start and Target Node	Test Items	Traditional A* Algorithm	A* Optimization Algorithm	Performance Optimization Rate
Long-distance planning	(6, 66), (30, 2)	Path nodes	78	11	85.9%
		Safe distance (m)	0	1	-
		Total turning angle (°)	800.1	601.3	24.8%
		Path length (m)	1303.5	1285.5	1.4%
Medium-distance planning	(17, 27), (3, 51)	Path nodes	28	5	82.1%
		Safe distance (m)	0	1	-
		Total turning angle (°)	405.3	234.7	42.1%
		Path length (m)	496.5	492	0.9%
Short-distance planning	(35, 61), (6, 65)	Path nodes	28	3	89.3%
		Safe distance (m)	0	1	-
		Total turning angle (°)	270.1	56.2	79.2%
		Path length (m)	460.5	447	2.9%

**Table 6:** Performance indicators of planning algorithms.

Test Items	Traditional A* Algorithm	A* Optimization Algorithm with Constant P	A* Optimization Algorithm with Dynamically Adjusted P
Path nodes	75	12	10
Safe distance (m)	0	1	1
Total turning angle (°)	855.1	522.3	574.6
Path length (m)	1314	1332	1421

## Data Availability

No data was used for the research described in the article.

## References

[1] Arab A., Yu K., Yu J., Yi J. (2024). Motion Planning and Control of Autonomous Aggressive Vehicle Maneuvers. IEEE Trans. Autom. Sci. Eng..

[2] Chen Y., Veer S., Karkus P., Pavone M. (2024). Interactive Joint Planning for Autonomous Vehicles. IEEE Robot. Autom. Lett..

[3] Wang D. (2012). Indoor mobile-robot path planning based on an improved A* algorithm. J. Tsinghua Univ. Sci. Technol..

[4] Liu Y., Pei X., Zhou H., Guo X. (2024). Spatiotemporal Trajectory Planning for Autonomous Vehicle based on Reachable Set and Iterative LQR. IEEE Trans. Veh. Technol..

[5] Zhang G., Hu X., Chai J. (2011). Summary of Path Planning Algorithm and Its Application. Mod. Mach..

[6] Pan H., Luo M., Wang J., Huang T., Sun W. (2024). A Safe Motion Planning and Reliable Control Framework for Autonomous Vehicles. IEEE Trans. Intell. Veh..

[7] Hadi B., Khosravi A., Sarhadi P. (2024). Adaptive Formation Motion Planning and Control of Autonomous Underwater Vehicles Using Deep Reinforcement Learning. IEEE J. Ocean. Eng..

[8] Wu Q., Chen Z., Wang L., Lin H., Jiang Z., Li S., Chen D. (2020). Real-Time Dynamic Path Planning of Mobile Robots: A Novel Hybrid Heuristic Optimization Algorithm. Sensors.

[9] Zhong Y.M., Shirinzadeh B., Yuan X.B. (2011). Optimal Robot Path Planning with Cellular Neural Network. Int. J. Intell. Mechatron. Robot..

[10] Hills J., Zhong Y. (2014). Cellular neural network-based thermal modelling for real-time robotic path planning. Int. J. Agil. Syst. Manag..

[11] Zhong Y.M., Shirinzadeh B., Tian Y.L. A New Neural Network for Robot Path Planning. Proceedings of the 2008 IEEE/ASME International Conference on Advanced Intelligent Mechatronics.

[12] Zhou Q., Gao S.S., Qu B.Y., Gao X., Zhong Y.M. (2022). Crossover recombination-based global-best brain storm optimization algorithm for UAV path planning. Proc. Rom. Acad. Ser. A Math. Phys. Technol. Sci. Inf. Sci..

[13] Karaman S., Frazzoli E. (2011). Sampling-based algorithms for optimal motion planning. Int. J. Robot. Res..

[14] Yu F., Shang H., Zhu Q., Zhang H., Chen Y. (2023). An efficient RRT-based motion planning algorithm for autonomous underwater vehicles under cylindrical sampling constraints. Auton. Robot..

[15] Umari H., Mukhopadhyay S. Autonomous Robotic Exploration Based on Multiple Rapidly-Exploring Randomized Trees. Proceedings of the 2017 IEEE/RSJ International Conference on Intelligent Robots and Systems (IROS).

[16] Kuffner J., LaValle S. RRT-connect: An efficient approach to single-query path planning. Proceedings of the 2000 ICRA. Millennium Conference. IEEE International Conference on Robotics and Automation. Symposia Proceedings (Cat. No.00CH37065).

[17] Karaman S., Frazzoli E. (2010). Incremental Sampling-based Algorithms for Optimal Motion Planning. Comput. Sci..

[18] Liao B., Hua Y., Wan F., Zhu S., Zong Y., Qing X. (2023). Stack-RRT*: A Random Tree Expansion Algorithm for Smooth Path Planning. Int. J. Control Autom. Syst..

[19] Christian Z., van Erik-Jan K. (2022). Comparison Between A* and RRT Algorithms for 3D UAV Path Planning. Unmanned Syst..

[20] Zhu B., Han J., Zhao J., Liu S., Deng W. (2020). Path planning method for intelligent vehicles based on improved RRT algorithm for safety field. Automot. Eng..

[21] Hsu D., Latombe J.-C., Kurniawati H. (2012). On the Probabilistic Foundations of Probabilistic Roadmap Planning. Int. J. Robot. Res..

[22] Elbanhawi M., Simic M. (2014). Sampling-Based Robot Motion Planning: A Review. IEEE Access.

[23] Hsu D., Latombe J.-C., Motwani R., Agarwal P.K. (1999). Path planning in expansive configuration spaces. Int. J. Comput. Geom. Appl..

[24] Long H., Li G., Tan X., Xue C., Yi J. (2024). Improved RRT robotic arm path planning by fusion A*. Comput. Eng. Appl..

[25] Osman I.H. (1993). Metastrategy simulated annealing and tabu search algorithms for the vehicle routing problem. Ann. Oper. Res..

[26] Ding S., Li C., Xu X., Ding L., Zhang J., Guo L., Shi T. (2023). A Sampling-Based Density Peaks Clustering Algorithm for Large-Scale Data. Pattern Recognit..

[27] Ma Y., Zhao Y., Li Z., Yan X., Bi H., Królczyk G. (2022). A new coverage path planning algorithm for unmanned surface mapping vehicle based on A-star based searching. Appl. Ocean Res..

[28] Wang H., Sun Z. (2011). Research on Multi-Constraint Multicast Routing Algorithm Based on Dijkstra Algorithm. Comput. Technol. Dev..

[29] Feng T., Li J., Jiang H., Yang S.X., Wang P., Teng Y., Chen S., Fu Q., Luo B. (2024). The Optimal Global Path Planning of Mobile Robot Based on Improved Hybrid Adaptive Genetic Algorithm in Different Tasks and Complex Road Environments. IEEE Access.

[30] Lian Y. (2011). Improved A* path planning algorithm for vision-guided multi-AGV system. Control. Decis..

[31] Yu W., Zhang Z., Fu X., Wang Z. (2020). Path planning based on map preprocessing and improved A* algorithm. High Tech Commun..

[32] Yao M., Deng H., Feng X., Li P., Li Y., Liu H. (2024). Improved dynamic windows approach based on energy consumption management and fuzzy logic control for local path planning of mobile robots. Comput. Ind. Eng..

[33] Cheng Y., Xiao H. (2020). Dynamic path planning of mobile robot fusing improved A* algorithm and Morphin algorithm. J. Intell. Syst..

[34] Chen Y., Wu J., He C., Zhang S. (2023). Intelligent Warehouse Robot Path Planning Based on Improved Ant Colony Algorithm. IEEE Access.

[35] Zhu B., Zhang J., Li J., Chen L., Wu J., Farisi Z. (2022). Path Planning of Energy Robot Based on Improved Ant Colony Algorithm. Wirel. Commun. Mob. Comput..

[36] Fransen K., van Eekelen J. (2023). Efficient path planning for automated guided vehicles using A* (Astar) algorithm incorporating turning costs in search heuristic. Int. J. Prod. Res..

[37] Wang P., Yang J., Zhang Y., Wang Q., Sun B., Guo D. (2022). Obstacle-Avoidance Path-Planning Algorithm for Autonomous Vehicles Based on B-Spline Algorithm. World Electr. Veh. J..

[38] Liao T., Chen F., Wu Y., Zeng H., Ouyang S., Guan J. (2024). Research on Path Planning with the Integration of Adaptive A-Star Algorithm and Improved Dynamic Window Approach. Electronics.

[39] Yu M., Luo Q., Wang H., Lai Y. (2023). Electric Logistics Vehicle Path Planning Based on the Fusion of the Improved A-Star Algorithm and Dynamic Window Approach. World Electr. Veh. J..

[40] Cui S., Chen Y., Li X. (2022). A Robust and Efficient UAV Path Planning Approach for Tracking Agile Targets in Complex Environments. Machines.

[41] Wang W., Pei D., Feng Z. (2018). The shortest path planning for mobile robots using improved A* algorithm. J. Comput. Appl..

[42] Duan S., Wang Q., Han X., Liu G. (2020). Improved A-star algorithm for safety insured optimal path with smoothed corner turns. J. Mech. Eng..

[43] Liu Z., Zhao J., Liu C. (2021). Path planning of indoor mobile robot based on improved A* algorithm. Comput. Eng. Appl..

[44] Shen K., You Z., Liu Y., Huang T. (2023). Path planning of Mobile Robot based on improved A* Algorithm. Appl. Res. Comput..

[45] Chen Y., Jiang W., Yang L., Luo Z. (2019). Path Planning for Mobile Robots Based on Motion Constraints. Comput. Integr. Manuf. Syst..

